# Systemic lupus erythematosus-related mortality declined but disparities persisted in the United States: a nationwide multiple-cause-of-death analysis, 1999–2023

**DOI:** 10.3389/fimmu.2026.1850485

**Published:** 2026-07-24

**Authors:** Yuhuan Song, Yan Zhang, Li Luo, Xiuju Yang, Kaide Xia

**Affiliations:** 1 Department of Nephrology, Aerospace Center Hospital, Beijing, China; 2 School of Public Health, Guizhou Medical University, Guiyang, China; 3 Department of Clinical Laboratory, The Second People’s Hospital of Guiyang, Guiyang, China; 4 Laboratory of Molecular Genetic Diagnostics, Guiyang Maternal and Child Health Care Hospital, Guiyang Children’s Hospital, Guiyang, China

**Keywords:** CDC WONDER, health disparities, immune dysregulation, mortality, multiple cause of death, systemic lupus erythematosus

## Abstract

**Background:**

Systemic lupus erythematosus (SLE) causes substantial premature mortality. Contemporary national data on long-term SLE-related mortality and its demographic and geographic disparities in the United States remain limited, particularly across the transition into the COVID-19 era.

**Methods:**

We conducted a nationwide observational study using the 1999–2023 CDC WONDER Multiple Cause of Death database. SLE-related mortality was defined as any death certificate mention of SLE (ICD-10 M32). An underlying-cause-only sensitivity analysis assessed trend robustness. Annual deaths and age-adjusted mortality rates (AAMRs) were examined overall and by sex, age, race, geography, and urbanization. Temporal trends were evaluated using joinpoint regression to estimate annual percent changes (APCs) and average annual percent changes (AAPCs). Pooled state-level AAMRs were compared between 2016–2019 and 2020–2023.

**Results:**

From 1999 to 2023, the national SLE-related AAMR declined from 1.03 to 0.73 per 100,000 (overall AAPC, −1.46%), while annual deaths increased slightly from 2,197 to 2,291. Joinpoint analysis identified a significant decline during 1999–2014, an increase during 2014–2021, and a sharp decline during 2021–2023. Women consistently had higher AAMRs than men. Adults aged ≥65 years had the highest mortality burden with little overall improvement. Black individuals maintained the highest AAMRs throughout, despite experiencing the steepest long-term decline. Geographically, the South, HHS Region 6, and Oklahoma remained high-burden areas. Nonmetropolitan areas showed smaller overall declines and steeper late-period increases. The underlying-cause-only sensitivity analysis also showed an AAMR decline from 0.59 to 0.29 per 100,000 (AAPC, −3.23%).

**Conclusion:**

U.S. SLE-related mortality declined overall from 1999 to 2023, but the absolute burden remained substantial and unequally distributed across demographic and geographic strata. Persistent excess mortality among women, older adults, Black individuals, and residents of high-burden areas highlights the need for more equitable translation of advances in lupus care into real-world survival gains.

## 1 Introduction

Systemic lupus erythematosus (SLE) is a prototypic systemic autoimmune disease characterized by loss of immune tolerance, autoantibody production, immune complex-driven inflammation, and progressive multi-organ injury (1–3). Although advances in diagnosis, monitoring, and immunomodulatory treatment have improved outcomes over time, SLE continues to impose substantial premature mortality (4–6). From the perspective of clinical and translational immunology, mortality patterns provide a population-level readout of the long-term consequences of immune dysregulation and the extent to which therapeutic progress has translated into survival benefit in real-world settings (2, 4).

The burden of SLE is not evenly distributed across populations (7, 8). Prior Centers for Disease Control and Prevention (CDC)-supported studies have shown that lupus disproportionately affects women and minority populations, and that Black individuals with SLE experience higher mortality and younger age at death than White individuals (8–11). These observations indicate that improvements in lupus care have not produced uniform gains across demographic groups and suggest that structural, clinical, and immunologic vulnerabilities may converge to shape outcome disparities (12–14).

Recent national work has described temporal trends in SLE-related mortality in the United States through 2022. However, further surveillance is needed to extend these findings through 2023 and to characterize whether demographic and geographic disparities persisted across the transition into the COVID-19 era. In particular, evidence remains limited regarding variation across Census regions, Department of Health and Human Services (HHS) regions, urbanization strata, and pooled state-level mortality patterns before and during the 2020–2023 period (11, 15). This question is important in the United States, where differences in access to subspecialty care, regional health-system resources, and broader social determinants may influence the downstream consequences of autoimmune disease. We therefore analyzed nationwide CDC Wide-ranging Online Data for Epidemiologic Research (WONDER) multiple-cause-of-death data from 1999 to 2023 to characterize long-term temporal trends in SLE-related mortality and to describe demographic and geographic disparities by sex, age, race, Census region, HHS region, urbanization, and pooled state-level periods. Our objectives were descriptive: to assess whether the national age-adjusted mortality rate declined over time and whether demographic and geographic inequities persisted (16).

## 2 Materials and methods

### 2.1 Study design and data source

We conducted a nationwide observational study using the Multiple Cause of Death database in CDC WONDER (17, 18). CDC WONDER mortality data are derived from U.S. death certificates and include one underlying cause of death, up to 20 additional multiple causes of death, and demographic information (18). In the analytic workflow used for this study, tab-delimited CDC WONDER extracts were imported recursively and harmonized into unified analytic datasets covering overall mortality and subgroup-specific analyses. The workflow incorporated final bridged-race files for earlier years and single-race files for later years, with harmonization into stable analytic categories for longitudinal comparison. This study used publicly available, de-identified, aggregate mortality data and did not involve direct participant contact or identifiable human information. Accordingly, ethics review and informed consent were not required.

### 2.2 Outcome definition and study variables

The primary outcome was any-mention SLE-related mortality, defined as deaths in which systemic lupus erythematosus (ICD-10 code M32 and its subcategories) was recorded anywhere on the death certificate, either as the underlying cause of death or as a contributing multiple cause of death. Accordingly, the primary outcome reflects mortality with SLE documented on the death certificate and does not necessarily indicate that SLE was the direct or immediate cause of death (4, 18). This multiple-cause-of-death definition was selected to capture the broader mortality burden associated with SLE, including deaths in which SLE may have contributed to fatal infection, cardiovascular disease, renal disease, malignancy, treatment-related complications, or cumulative organ damage. However, this approach cannot determine the relative contribution of SLE to each death, distinguish direct SLE-attributable deaths from deaths primarily due to other conditions, or estimate the attributable fraction of mortality due to SLE. Because SLE may not be recorded on some death certificates even among individuals with clinically established disease, the observed rates may underestimate the true mortality burden associated with SLE. Annual deaths, age-adjusted mortality rates (AAMRs), and corresponding 95% confidence intervals were examined at the national level and across predefined strata. Demographic subgroup analyses included sex (female and male), age group (15–44, 45–64, and ≥65 years), and race (White, Black, and Other). To preserve comparability across earlier bridged-race mortality files and later single-race files, and to reduce instability in annual estimates for smaller groups, race was analyzed using these harmonized broad categories. The Other category combined heterogeneous populations and therefore should not be interpreted as a single epidemiologically homogeneous group. Hispanic ethnicity was not analyzed independently in the present study. Geographic disparities were evaluated across three established administrative and demographic stratifications: the four United States Census Bureau regions (19), the ten Department of Health and Human Services (HHS) regions (20), and urbanization levels categorized according to the 2013 National Center for Health Statistics (NCHS) Urban-Rural Classification Scheme for Counties (21). Because urbanization-specific estimates in the available CDC WONDER extracts were only reported through 2020, analyses by urbanization were limited to 1999–2020. All analyses were restricted to decedents aged 15 years or older, consistent with the prespecified age-stratified categories of 15–44, 45–64, and ≥65 years.

### 2.3 Statistical analysis

AAMR was the primary rate-based endpoint. Temporal trends in annual AAMRs were analyzed using the Joinpoint Regression Program, Version 5.4.0.0 (April 2025; Statistical Research and Applications Branch, National Cancer Institute), which was invoked from R (version 4.5.2). R was used to prepare annual trend datasets, batch-submit Joinpoint analyses, import output files, and generate figures (22).

For each national and subgroup-specific annual series, calendar year was specified as the independent variable and the natural logarithm of the annual AAMR was specified as the dependent variable. Candidate log-linear models with 0 to 2 joinpoints were evaluated. A maximum of 2 joinpoints was prespecified to avoid overfitting and to preserve interpretability given the available annual observations. The final model was selected using the Monte Carlo permutation test with the program default settings and a two-sided significance level of 0.05. Models were fitted without assuming constant variance. Annual percent changes (APCs) were estimated for each identified trend segment, and average annual percent changes (AAPCs) were calculated over the full available period for each series. Ninety-five percent confidence intervals were calculated for APCs and AAPCs.

No formal adjustment for multiple comparisons was applied because the overall and subgroup analyses were descriptive and based on prespecified demographic and geographic strata. Accordingly, APC and AAPC P values were interpreted cautiously together with 95% confidence intervals, effect sizes, and consistency of temporal patterns across related strata.

As a sensitivity analysis, we repeated the national temporal trend analysis using a narrower underlying-cause-only definition of SLE mortality. In this analysis, deaths were included only when SLE (ICD-10 codes M32.0, M32.1, M32.8, or M32.9) was listed as the underlying cause of death. Annual AAMRs were calculated using the same standard population and were analyzed using the same Joinpoint modeling approach as in the primary analysis. This analysis was performed to assess whether the overall national mortality trend was robust to defining SLE as the underlying cause rather than as any mention on the death certificate.

To reduce instability arising from suppressed or unreliable annual state-level estimates, state-level analyses were performed using pooled AAMRs for two multi-year periods, 2016–2019 and 2020–2023, and absolute and relative changes between periods were calculated. This pooling strategy was used to improve the robustness and interpretability of subnational comparisons.

## 3 Results

### 3.1 National overall temporal trends

**Figure 1:**
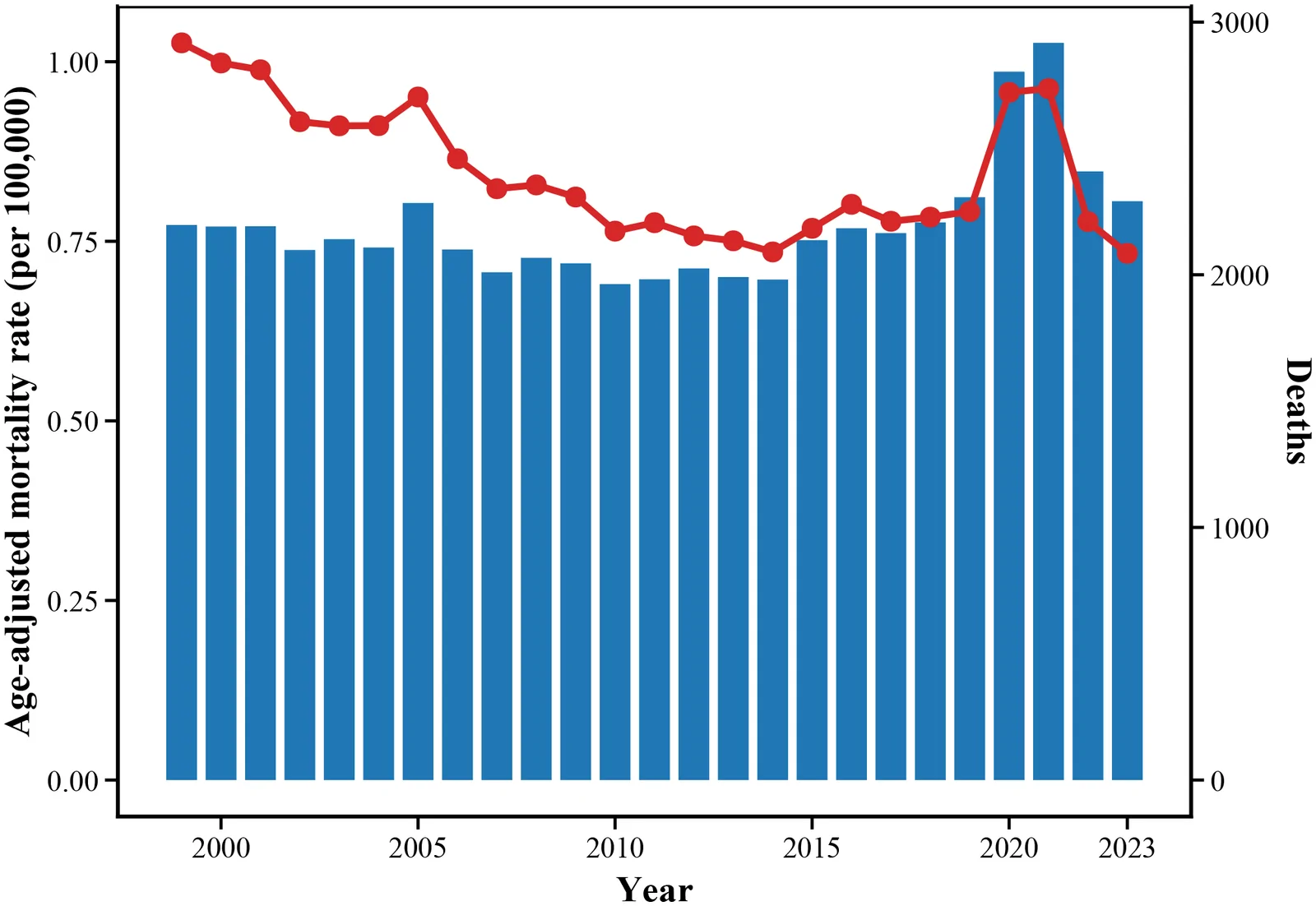
Annual deaths and AAMR for SLE-related mortality in the United States, 1999–2023. Blue bars represent annual numbers of deaths on the right y-axis, and the red line with points represents the age-adjusted mortality rate per 100,000 population on the left y-axis. AAMR, age-adjusted mortality rate; SLE, systemic lupus erythematosus.

From 1999 to 2023, the national age-adjusted mortality rate (AAMR) for SLE-related mortality in the United States declined overall, whereas annual deaths fluctuated over time rather than showing a sustained monotonic decrease. The AAMR decreased from the early study period to the mid-2010s, rose during the late pre-pandemic and pandemic-transition years, and then declined again after 2021, while the absolute number of deaths remained substantial throughout the study period (**Figure 1**).

Consistently, the national number of SLE-related deaths increased slightly from 2,197 in 1999 to 2,291 in 2023, whereas the national AAMR declined from 1.03 (95% CI, 0.98–1.07) to 0.73 (95% CI, 0.70–0.76). The AAPC over the full study period was −1.46% (95% CI, −1.88 to −1.13). Joinpoint analysis identified three phases in the national AAMR trajectory: a significant decline during 1999–2014, an increase during 2014–2021, and a sharp decline during 2021–2023, with corresponding APCs of −2.27%, 3.62%, and −12.08%, respectively (**Table 1**).

**Table 1:** Trends and characteristics of SLE-related mortality in the United States, 1999–2023.

Group	1999		2023		AAPC (95% CI)
	Deaths	AAMR (95% CI)	Deaths	AAMR (95% CI)	
Overall	2197	1.03 (0.98 to 1.07)	2291	0.73 (0.70 to 0.76)	-1.46 (-1.88 to -1.13)
Sex					
Female	1,852	1.58 (1.51 to 1.65)	1,924	1.19 (1.13 to 1.24)	-1.39 (-1.85 to -0.96)
Male	345	0.36 (0.33 to 0.40)	367	0.25 (0.22 to 0.28)	-1.39 (-2.2 to -0.57)
Age, years					
15-44	643	0.55 (0.51 to 0.59)	354	0.27 (0.25 to 0.30)	-2.77 (-3.32 to -2.39)
45-64	711	1.18 (1.09 to 1.27)	666	0.78 (0.72 to 0.84)	-1.92 (-2.68 to -1.44)
≥65	843	2.41 (2.25 to 2.57)	1,271	2.24 (2.12 to 2.37)	0.08 (-0.41 to 0.58)
Race					
White	1,468	0.79 (0.75 to 0.83)	1,539	0.58 (0.55 to 0.61)	-1.26 (-1.92 to -0.88)
Black	643	2.61 (2.40 to 2.81)	607	1.65 (1.52 to 1.79)	-2.23 (-2.98 to -1.65)
Other	86	0.91 (0.71 to 1.14)	121	0.53 (0.44 to 0.64)	-1.9 (-3.04 to -0.27)
Urbanization^a^					
Metropolitan	1,820	1.01 (0.96 to 1.05)	2,379	0.96 (0.92 to 0.99)	-0.77 (-1.19 to -0.32)
Nonmetropolitan	377	1.01 (0.91 to 1.11)	425	0.93 (0.84 to 1.02)	-0.43 (-1.41 to 0.16)

^a^ Urbanization-specific estimates were available only through 2020 in CDC WONDER. Accordingly, the values shown in the final-year columns for the urbanization rows represent estimates for 2020 rather than 2023. AAMR, age-adjusted mortality rate; SLE, systemic lupus erythematosus; AAPC, average annual percent change.

In the underlying-cause-only sensitivity analysis, national SLE mortality rates were lower than those observed using the primary any-mention multiple-cause definition. The AAMR declined from 0.59 per 100,000 (95% CI, 0.56–0.63) in 1999 to 0.29 per 100,000 (95% CI, 0.27–0.31) in 2023, corresponding to an AAPC of −3.23% (95% CI, −4.04 to −2.54). Joinpoint analysis identified a significant decline from 1999 to 2021 (APC, −2.18%; 95% CI, −2.52 to −1.51), followed by a steeper decline from 2021 to 2023 (APC, −14.05%; 95% CI, −22.49 to −3.81). Although the narrower underlying-cause-only definition supported the overall long-term decline in SLE mortality, it did not reproduce the intermediate increase observed in the primary any-mention analysis during 2014–2021. These findings indicate that the long-term downward trend was consistent across outcome definitions, whereas the short-term segment-specific pattern was sensitive to whether SLE was recorded as the underlying cause or as any contributing cause of death (**Supplementary Table 3**, **Supplementary Figure 4**).

### 3.2 Demographic disparities

**Figure 2:**
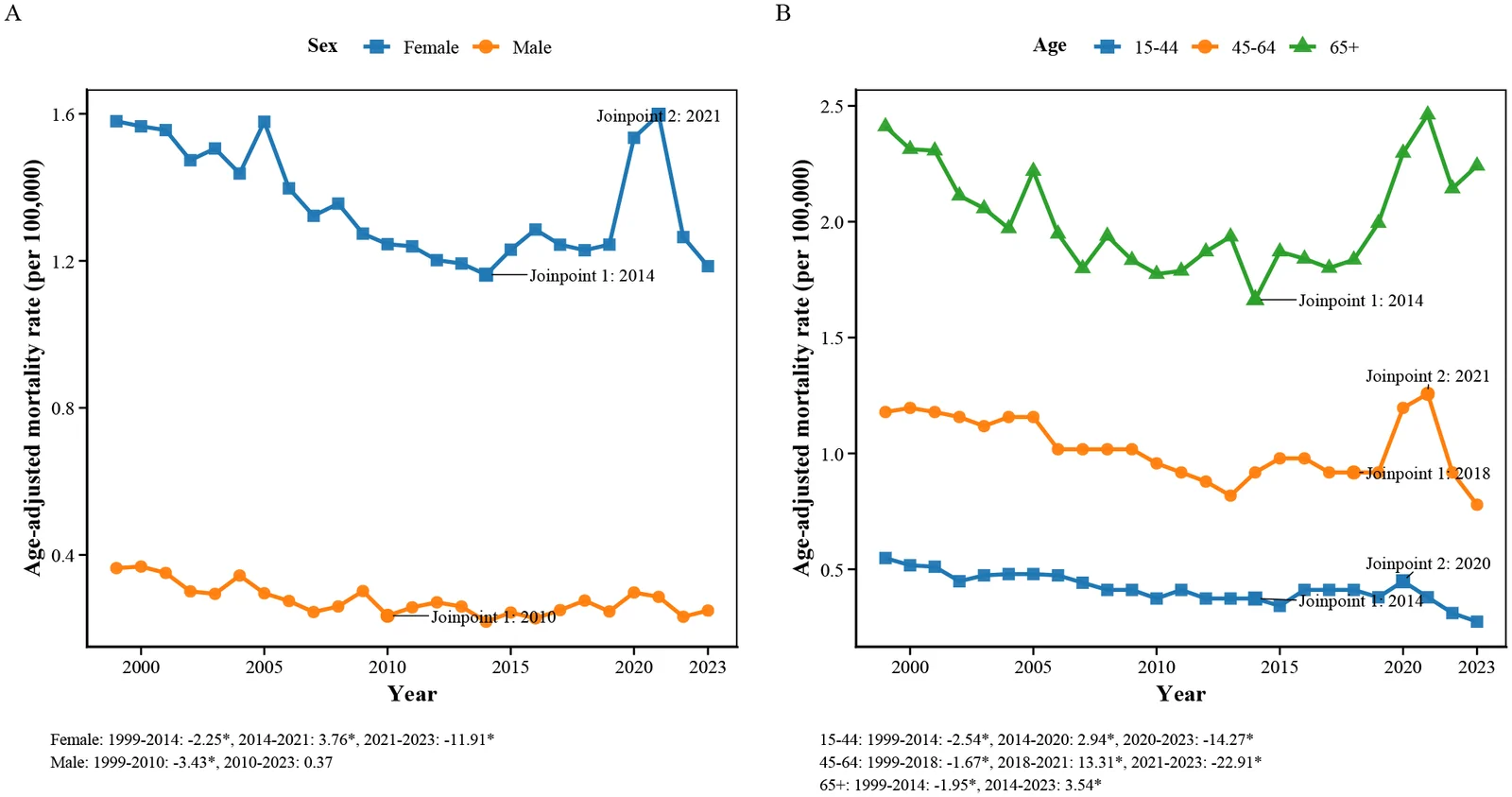
Trends in sex- and age-specific SLE-related AAMR in the United States, 1999–2023. **(A)** Sex. **(B)** Age group. *Indicate APCs significantly different from zero at P < 0.05. SLE, systemic lupus erythematosus; AAMR, age-adjusted mortality rate.

Marked demographic disparities in SLE-related mortality were observed across sex, age group, and race. Women consistently had substantially higher AAMRs than men throughout the study period. The number of SLE-related deaths in women increased slightly from 1,852 in 1999 to 1,924 in 2023, whereas the AAMR declined from 1.58 (95% CI, 1.51–1.65) to 1.19 (95% CI, 1.13–1.24). In men, deaths increased from 345 to 367, while the AAMR decreased from 0.36 (95% CI, 0.33–0.40) to 0.25 (95% CI, 0.22–0.28). The overall AAPCs were identical in women and men (both −1.39%), although the temporal patterns differed; women experienced a significant decline during 1999–2014, followed by an increase during 2014–2021 and a sharp decline during 2021–2023, whereas men showed an early decline during 1999–2010 and relative stability thereafter (**Table 1**, **Figure 2A**).

Age-stratified analyses also demonstrated substantial heterogeneity. The ≥65-year group consistently had the highest AAMRs over the entire study period, followed by the 45–64-year group and the 15–44-year group. From 1999 to 2023, deaths declined in the 15–44-year group (643 to 354) and the 45–64-year group (711 to 666), but increased markedly in the ≥65-year group (843 to 1,271). Over the same period, the AAMR decreased from 0.55 to 0.27 in the 15–44-year group and from 1.18 to 0.78 in the 45–64-year group, while remaining comparatively high in the ≥65-year group, changing only modestly from 2.41 to 2.24. Consistently, the AAPC was most negative in the 15–44-year group (−2.77%), followed by the 45–64-year group (−1.92%), whereas the ≥65-year group showed no clear overall decline (AAPC: 0.08%). The 15–44-year and 45–64-year groups both exhibited a late-period rebound before declining again after 2020 or 2021, whereas the ≥65-year group declined initially but increased during the later years (**Table 1**, **Figure 2B**).

Race-specific analyses revealed persistent and pronounced disparities. Black individuals had the highest AAMRs throughout the study period, substantially exceeding those of White and Other racial groups. In 1999, the AAMR was 2.61 (95% CI, 2.40–2.81) in Black individuals, compared with 0.79 (95% CI, 0.75–0.83) in White individuals and 0.91 (95% CI, 0.71–1.14) in the Other racial group; by 2023, the corresponding AAMRs were 1.65 (95% CI, 1.52–1.79), 0.58 (95% CI, 0.55–0.61), and 0.53 (95% CI, 0.44–0.64), respectively. The overall decline was steepest in Black individuals (AAPC: −2.23%), compared with −1.26% in White individuals and −1.90% in the Other racial group. Black individuals maintained the highest mortality burden across all years, with a decline during 1999–2015, an increase during 2015–2021, and a sharp decline after 2021. White individuals showed a similar but less pronounced rebound during 2017–2021, whereas the Other racial group declined before 2010 and remained broadly stable thereafter (**Table 1**, **Supplementary Figure 1**).

### 3.3 Geographic disparities

**Figure 3:**
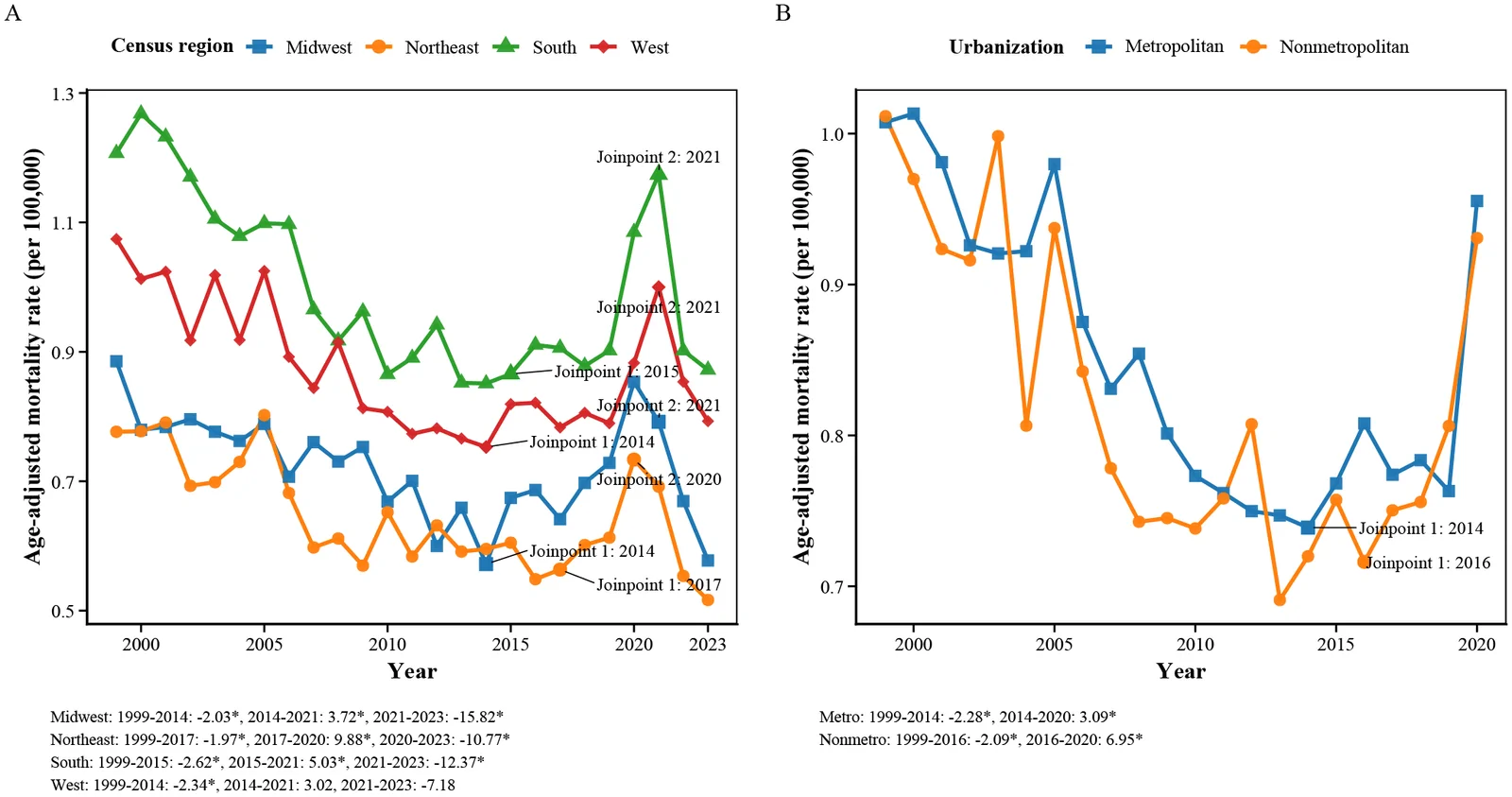
Trends in geographically stratified SLE-related AAMRs in the United States. **(A)** Census region, 1999–2023. **(B)** Urbanization, 1999–2020. Urbanization-specific estimates were available only through 2020 in the extracted CDC WONDER data. Asterisks indicate APCs significantly different from zero at P < 0.05. AAMR, age-adjusted mortality rate; APC, annual percent change; SLE, systemic lupus erythematosus.

Clear geographic disparities in SLE-related mortality were observed across both broad regions and urbanization strata. At the Census-region level, the South consistently had the highest mortality burden throughout the study period, whereas the Northeast remained the lowest. From 1999 to 2023, the AAMR decreased from 1.21 (95% CI, 1.13–1.28) to 0.87 (95% CI, 0.82–0.93) in the South, from 1.07 (95% CI, 0.98–1.17) to 0.79 (95% CI, 0.73–0.86) in the West, from 0.89 (95% CI, 0.80–0.97) to 0.58 (95% CI, 0.52–0.64) in the Midwest, and from 0.78 (95% CI, 0.69–0.86) to 0.52 (95% CI, 0.46–0.58) in the Northeast. Over the same period, deaths increased in the South (922 to 1,028) and West (492 to 573), but declined in the Midwest (439 to 388) and Northeast (344 to 302). All four Census regions experienced an early decline followed by a later rebound before 2020 or 2021, with the South maintaining the highest AAMRs across nearly the entire study period. The overall AAPCs ranged from −1.22% in the West to −1.73% in the Northeast, indicating persistent but uneven regional improvement (**Supplementary Table 1**, **Figure 3A**).

Urbanization-stratified analyses also showed heterogeneity. Both metro and nonmetro areas had comparable baseline AAMRs in 1999 (both 1.01), but by 2020 the AAMR remained slightly higher in metro areas [0.96 (95% CI, 0.92–0.99)] than in nonmetro areas [0.93 (95% CI, 0.84–1.02)]. Absolute deaths increased from 1,820 to 2,379 in metro areas and from 377 to 425 in nonmetro areas. Both strata experienced long-term declines followed by renewed increases in the later years available for this analysis, with a steeper late-period increase in nonmetro areas. The corresponding AAPCs were −0.77% for metro areas and −0.43% for nonmetro areas, suggesting a more modest overall decline in nonmetro settings (**Table 1**, **Figure 3B**).

Patterns across HHS regions broadly supported substantial subnational heterogeneity. HHS Region 6 had the highest AAMRs at both the beginning and end of the study period, declining from 1.41 (95% CI, 1.26–1.56) in 1999 to 1.00 (95% CI, 0.90–1.11) in 2023, whereas HHS Region 1 remained among the lowest [0.51 (95% CI, 0.39–0.66) in 1999 and 0.50 (95% CI, 0.40–0.63) in 2023]. HHS Region 4 also showed a persistently elevated burden, with deaths increasing from 473 to 507 despite a decline in AAMR from 1.12 to 0.75. Temporal patterns varied substantially across HHS regions, with several regions showing long-term declines interrupted by rebounds before 2021, followed by renewed declines thereafter (**Supplementary Table 1**, **Supplementary Figure 2**).

### 3.4 State-level pooled spatial comparison

**Figure 4:**
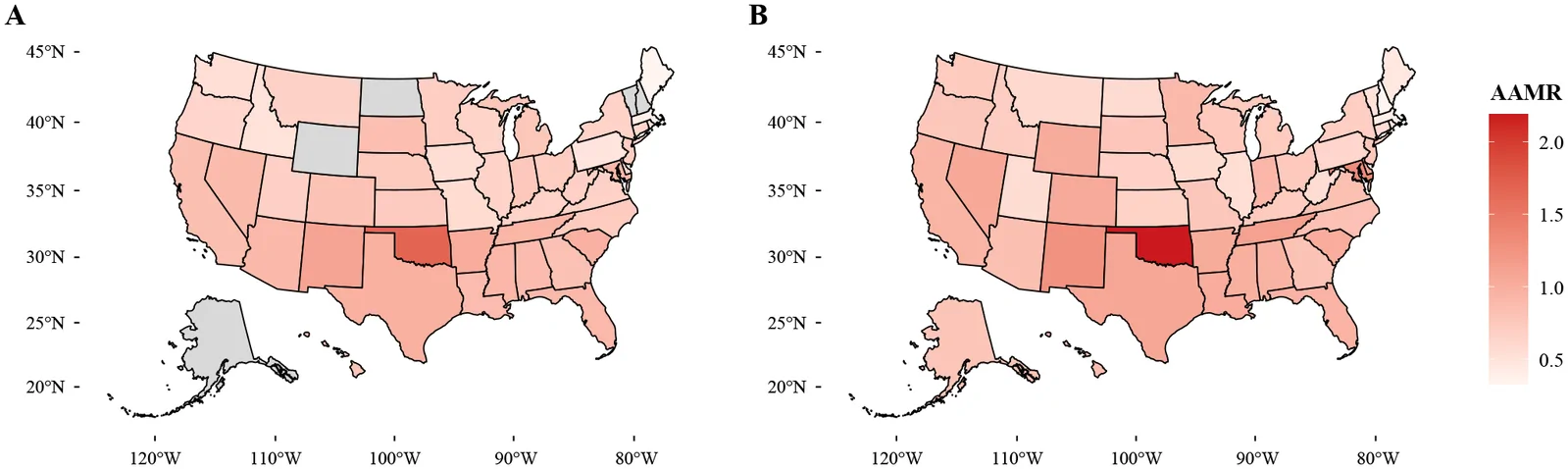
State-level pooled SLE-related age-adjusted mortality rates in the United States, 2016–2019 and 2020–2023. **(A)** 2016–2019. **(B)** 2020–2023. Gray indicates states with unavailable or unreliable pooled AAMR estimates. AAMR, age-adjusted mortality rate; SLE, systemic lupus erythematosus.

To reduce instability from suppressed or unreliable annual state-level estimates, pooled state-level AAMRs were compared between 2016–2019 and 2020–2023. Substantial geographic heterogeneity was observed in both periods. In the pre-COVID period, relatively higher pooled AAMRs were observed in parts of the South and selected central states, whereas lower rates were generally seen in parts of the Northeast and Upper Midwest. In the COVID-era period, the overall spatial pattern was broadly preserved, but several states exhibited higher pooled AAMRs than in the earlier period. Oklahoma remained among the states with the highest pooled AAMRs in both periods and showed particularly prominent elevation in 2020–2023 (**Figure 4**).

The change map further demonstrated that shifts in pooled mortality burden were spatially heterogeneous rather than uniform across the country. The largest absolute increases in pooled AAMR were concentrated in selected central and southern states, with Oklahoma showing the most pronounced increase. In contrast, several states in the interior West and central United States showed relatively small changes or modest declines (**Supplementary Figure 3**).

## 4 Discussion

In this nationwide analysis, the U.S. AAMR for SLE-related mortality declined overall between 1999 and 2023, whereas the annual number of deaths remained substantial. Importantly, the temporal pattern was not monotonic. National mortality fell from the early study period to the mid-2010s, increased during the late pre-pandemic and pandemic-transition years, and then declined again after 2021. This overall improvement coexisted with marked inequality: women had substantially higher AAMRs than men, adults aged ≥65 years had the highest and least improved mortality burden, Black individuals consistently had the highest mortality rates, and the South, HHS Region 6, and selected states—particularly Oklahoma—remained high-burden settings.

The decline in age-adjusted mortality rates despite persistently substantial annual death counts is not contradictory. AAMRs account for changes in population age structure, whereas the absolute number of deaths is influenced by population growth, population aging, changes in the number of people living with SLE, and survival into older age. Thus, improvements in age-adjusted mortality can coexist with a sustained or increasing absolute mortality burden. The present ecological mortality data cannot partition the relative contributions of demographic change, disease prevalence, diagnostic recognition, survival, and treatment advances to the observed death counts.

The overall decline in SLE-related mortality is likely multifactorial, but it may in part reflect major therapeutic advances in lupus care over the past two decades (23, 24). In particular, the broader adoption of immunosuppressive agents such as mycophenolate mofetil in the 2000s, especially in the management of lupus nephritis (25–28), and the FDA approval of belimumab in 2011, the first biologic approved specifically for SLE (29–31), may have contributed to improved disease control and long-term survival at the population level (23, 24).

From an immunology perspective, these findings suggest that the long-term clinical consequences of immune dysregulation remain unevenly distributed across populations and regions. The persistent excess burden in women is consistent with the strong female predominance of SLE (32, 33). The sustained excess mortality in Black individuals aligns with CDC-supported evidence and more recent syntheses showing higher mortality among underserved racial and ethnic groups with SLE (10). These racial and geographic patterns are descriptive and should not be interpreted as causal effects of race, ethnicity, or place. They may reflect multiple interrelated biological, social, structural, healthcare, and measurement-related factors, including differences in disease severity, cumulative organ damage, comorbidity, infection risk, treatment exposure, insurance coverage, access to subspecialty care, diagnostic recognition, and death certificate reporting. Because CDC WONDER does not include these individual-level variables, residual confounding and unmeasured contextual factors remain substantial limitations (34–37).

Age-specific findings warrant particular attention. Adults aged ≥65 years had the highest AAMRs throughout the study period and showed little overall improvement, whereas younger age groups experienced clearer declines. These descriptive patterns should not be interpreted as evidence that age itself is a causal determinant of SLE-related mortality. Rather, older adults with SLE may accumulate longstanding immune-mediated organ injury, chronic kidney disease, cardiovascular disease, frailty, multimorbidity, infection susceptibility, medication-related toxicity, and competing late-life risks (38–41). These factors may reduce the extent to which advances in lupus diagnosis and treatment translate into mortality improvement among older patients.

In addition, the present multiple-cause-of-death design cannot determine whether SLE, cardiovascular disease, infection, malignancy, renal disease, or other conditions were the immediate causes of death in older decedents. Death certificate data also do not allow assessment of whether SLE was consistently recorded among older decedents with complex multimorbidity. Therefore, the persistent burden in adults aged ≥65 years should be interpreted as a high-risk descriptive pattern requiring more detailed cohort-based investigation rather than as evidence of a specific causal mechanism.

By contrast, the more favorable long-term declines observed in younger and middle-aged adults may be compatible with earlier diagnosis, improved monitoring, and more standardized long-term disease management (6, 42). However, these potential explanations could not be evaluated directly in the present dataset.

The apparent increase in any-mention SLE-related mortality during the later years of the primary analysis should be interpreted cautiously. In the underlying-cause-only sensitivity analysis, the long-term decline persisted through 2021 and no corresponding intermediate increase was observed. This difference indicates that short-term fluctuations in the any-mention series may reflect not only changes in deaths directly attributable to SLE, but also changes in the documentation of SLE as a contributing condition, shifts in comorbidity patterns, or variation in the clinical circumstances surrounding death.

The temporal overlap between the later study period and the COVID-19 era raises clinically plausible hypotheses, including increased infection-related vulnerability, disruption of routine care, delayed monitoring, reduced access to specialty services, and challenges related to immunosuppressive therapy (5, 15, 43). However, CDC WONDER does not provide individual-level information on SARS-CoV-2 infection, COVID-19 vaccination, treatment interruption, disease activity, healthcare utilization, or clinical causes of death. Therefore, this study cannot determine whether COVID-19 directly contributed to the observed temporal changes, quantify the contribution of healthcare disruption, or distinguish pandemic-related effects from changes in death certificate reporting or pre-existing mortality patterns. These observations should therefore be considered hypothesis-generating rather than causal.

In a broader international context, the overall decline in U.S. age-adjusted SLE-related mortality is broadly compatible with improvements in survival reported in several high-income settings (6, 44). However, direct numerical comparison across countries is difficult because studies differ substantially in SLE case definitions, ascertainment methods, death certificate practices, underlying-cause versus multiple-cause mortality definitions, access to specialty care, and population composition (8). Global reviews continue to show marked geographic variation in SLE burden and persistent excess mortality compared with the general population (8). Thus, the present U.S. findings should be interpreted as one national surveillance perspective rather than as directly comparable estimates of worldwide SLE mortality.

Several limitations should be acknowledged. First, this study used an any-mention multiple-cause-of-death definition of SLE-related mortality. This approach captures deaths in which SLE was recorded as either an underlying or contributing cause, but it cannot determine whether SLE was the direct cause of death, quantify its contribution relative to other listed conditions, or estimate SLE-attributable mortality. Conversely, SLE may not be recorded on some death certificates, which could lead to underestimation of the mortality burden. Second, death certificate completion, diagnostic recognition, coding practices, and the likelihood of recording SLE as a contributing condition may have changed over time. Although ICD-10 M32 was used consistently to define SLE-related mortality, secular changes in reporting practices could have influenced observed temporal trends. Third, CDC WONDER does not provide patient-level information on disease duration, organ involvement, disease activity, autoantibody profile, treatment exposure, comorbidity, socioeconomic circumstances, insurance coverage, access to rheumatology care, or healthcare utilization. Therefore, residual confounding and unmeasured clinical and structural factors may substantially contribute to the observed demographic and geographic disparities. Fourth, race categories were broad, the other category combined heterogeneous populations, and Hispanic ethnicity was not analyzed independently. Fifth, harmonization across bridged-race and later single-race files may have introduced residual comparability challenges. Sixth, urbanization-specific estimates were available only through 2020. Finally, the analyses were descriptive and observational; they cannot establish causal mechanisms linking sex, age, race, geography, COVID-19-era conditions, healthcare access, or treatment advances to mortality trends.

## 5 Conclusions

Overall, SLE-related mortality in the United States declined over the last quarter century, but the benefits were incomplete and unequally distributed. The persistent concentration of mortality burden among older adults, women, Black individuals, and high-burden regions indicates that future progress will depend not only on continued advances in lupus immunopathogenesis and therapy, but also on more equitable translation of those advances into real-world care. Future work should link national mortality surveillance with cohort-level clinical validation, treatment data, and immunologic phenotyping to better explain why these mortality gaps persist.

## Data Availability

Publicly available datasets were analyzed in this study. These data can be found in the CDC WONDER Multiple Cause of Death database (https://wonder.cdc.gov/mcd.html). The processed results supporting the conclusions of this article are included in the article/Supplementary Material; further inquiries can be directed to the corresponding author.
