# Correction: Systemic lupus erythematosus-related mortality declined but disparities persisted in the United States: a nationwide multiple-cause-of-death analysis, 1999–2023

**DOI:** 10.3389/fimmu.2026.1955024

**Published:** 2026-08-12

**Authors:** Yuhuan Song, Yan Zhang, Li Luo, Xiuju Yang, Kaide Xia

**Affiliations:** 1 Department of Nephrology, Aerospace Center Hospital, Beijing, China; 2 School of Public Health, Guizhou Medical University, Guiyang, China; 3 Department of Clinical Laboratory, The Second People’s Hospital of Guiyang, Guiyang, China; 4 Laboratory of Molecular Genetic Diagnostics, Guiyang Maternal and Child Health Care Hospital, Guiyang Children’s Hospital, Guiyang, China

**Keywords:** CDC WONDER, health disparities, immune dysregulation, mortality, multiple cause of death, systemic lupus erythematosus

In the published article, the author names, author order, equal-contribution designation, and corresponding-author designation were correct, but the affiliation superscripts were incorrectly assigned. The correct author list reads:

“Yuhuan Song¹†, Yan Zhang²†, Li Luo³†, Xiuju Yang^4^, Kaide Xia^4^*”

There was an error in the **Supplementary Material** as published. The published file contained incorrect author information, which has been removed from the corrected version. The corrected **Supplementary Material** has been uploaded accordingly.

The original version of this article has been updated.

