# Supplementary material for: Systemic lupus erythematosus-related mortality declined but disparities persisted in the United States: a nationwide multiple-cause-of-death analysis, 1999–2023
Source: Front Immunol. 2026 Jul 24;17:1850485. doi: 10.3389/fimmu.2026.1850485 (PMC13447312; doi:10.3389/fimmu.2026.1850485)
Supplement: Supplementary file 1 [file Table1.docx]

**Supplementary to:**

**Systemic lupus erythematosus-related mortality declined but disparities persisted in the United States: a nationwide multiple-cause-of-death analysis, 1999–2023**

**Content**

[**Supplementary Table:**	2](#_Toc236437989)

[**Table S1.**	2](#_Toc236437990)

[**Table S2.**	2](#_Toc236437991)

[**Table S3.**	4](#_Toc236437992)

[**Supplementary Figure:**	5](#_Toc236437993)

[**Figure S1.**	5](#_Toc236437994)

[**Figure S2.**	6](#_Toc236437995)

[**Figure S3.**	7](#_Toc236437996)

[**Figure S4.**	8](#_Toc236437997)

# **Supplementary Table:**

| **Table S1. Trends and characteristics of SLE-related mortality by Census region and HHS region in the United States, 1999–2023.** | | | | | | |
| --- | --- | --- | --- | --- | --- | --- |
| **Group** | **1999** | |  | **2023** | | **AAPC (95% CI)** |
|  | **Deaths** | **AAMR (95% CI)** |  | **Deaths** | **AAMR (95% CI)** |  |
| **Census region** | |  |  |  |  |  |
| Northeast | 344 | 0.78 (0.69 to 0.86) |  | 302 | 0.52 (0.46 to 0.58) | -1.73 (-2.45 to -1.19) |
| Midwest | 439 | 0.89 (0.80 to 0.97) |  | 388 | 0.58 (0.52 to 0.64) | -1.64 (-2.45 to -1.12) |
| South | 922 | 1.21 (1.13 to 1.28) |  | 1,028 | 0.87 (0.82 to 0.93) | -1.63 (-2.3 to -1.15) |
| West | 492 | 1.07 (0.98 to 1.17) |  | 573 | 0.79 (0.73 to 0.86) | -1.22 (-1.76 to -0.55) |
| **HHS region** | |  |  |  |  |  |
| HHS Region 1 | 58 | 0.51 (0.39 to 0.66) |  | 77 | 0.50 (0.40 to 0.63) | -0.66 (-1.59 to 0.3) |
| HHS Region 10 | 71 | 0.85 (0.66 to 1.07) |  | 103 | 0.74 (0.60 to 0.91) | 0.02 (-1.15 to 0.94) |
| HHS Region 2 | 197 | 0.90 (0.77 to 1.03) |  | 160 | 0.59 (0.50 to 0.69) | -0.98 (-1.74 to -0.25) |
| HHS Region 3 | 206 | 0.91 (0.79 to 1.04) |  | 234 | 0.78 (0.68 to 0.89) | -0.56 (-1.41 to 0.25) |
| HHS Region 4 | 473 | 1.12 (1.02 to 1.22) |  | 507 | 0.75 (0.68 to 0.83) | -2.01 (-2.87 to -1.52) |
| HHS Region 5 | 345 | 0.90 (0.80 to 0.99) |  | 304 | 0.62 (0.56 to 0.70) | -1.24 (-2.09 to -0.74) |
| HHS Region 6 | 341 | 1.41 (1.26 to 1.56) |  | 371 | 1.00 (0.90 to 1.11) | -1.49 (-2.07 to -1.08) |
| HHS Region 7 | 89 | 0.85 (0.68 to 1.05) |  | 71 | 0.52 (0.41 to 0.67) | -2.06 (-3.27 to -0.82) |
| HHS Region 8 | 56 | 0.83 (0.62 to 1.07) |  | 85 | 0.79 (0.63 to 0.97) | -0.59 (-1.74 to 0.53) |
| HHS Region 9 | 361 | 1.17 (1.05 to 1.29) |  | 379 | 0.81 (0.73 to 0.90) | -1.48 (-2.48 to 0.01) |

Abbreviations: AAMR, age-adjusted mortality rate; SLE, systemic lupus erythematosus; AAPC, average annual percent change; HHS, Department of Health and Human Services.

| **Table S2. Trends and characteristics of state-level pooled SLE-related mortality in the United States, 2016–2019 and 2020–2023.** | | | | | | | |
| --- | --- | --- | --- | --- | --- | --- | --- |
| **State** | **2016-2019** | |  | **2020-2023** | | **Absolute change in AAMR** | **Percent change in AAMR (%)** |
|  | **Deaths** | **AAMR (95% CI)** |  | **Deaths** | **AAMR (95% CI)** |  |  |
| Alabama | 155 | 0.88 (0.74 to 1.02) |  | 177 | 0.95 (0.81 to 1.11) | 0.07 | 8.35 |
| Alaska | 13 | NA |  | 21 | 0.79 (0.48 to 1.27) | NA | NA |
| Arizona | 226 | 0.90 (0.78 to 1.02) |  | 223 | 0.84 (0.73 to 0.96) | -0.07 | -7.45 |
| Arkansas | 108 | 1.02 (0.82 to 1.22) |  | 115 | 1.03 (0.85 to 1.25) | 0.01 | 1.31 |
| California | 1,165 | 0.84 (0.79 to 0.89) |  | 1,298 | 0.94 (0.89 to 0.99) | 0.1 | 11.43 |
| Colorado | 163 | 0.81 (0.68 to 0.94) |  | 210 | 1.04 (0.90 to 1.19) | 0.23 | 27.87 |
| Connecticut | 83 | 0.62 (0.49 to 0.78) |  | 83 | 0.58 (0.46 to 0.73) | -0.05 | -7.25 |
| Delaware | 24 | 0.63 (0.40 to 0.94) |  | 43 | 1.15 (0.82 to 1.58) | 0.52 | 82.14 |
| District of Columbia | 25 | 1.27 (0.81 to 1.88) |  | 26 | 1.13 (0.73 to 1.68) | -0.14 | -11 |
| Florida | 710 | 0.90 (0.83 to 0.97) |  | 856 | 0.96 (0.89 to 1.03) | 0.06 | 6.19 |
| Georgia | 275 | 0.82 (0.72 to 0.92) |  | 291 | 0.81 (0.72 to 0.91) | -0.01 | -1.31 |
| Hawaii | 43 | 0.80 (0.57 to 1.08) |  | 48 | 0.86 (0.62 to 1.16) | 0.06 | 7.98 |
| Idaho | 30 | 0.51 (0.34 to 0.73) |  | 52 | 0.71 (0.53 to 0.94) | 0.2 | 38.83 |
| Illinois | 291 | 0.65 (0.57 to 0.73) |  | 246 | 0.55 (0.48 to 0.62) | -0.1 | -16.1 |
| Indiana | 181 | 0.75 (0.64 to 0.86) |  | 221 | 0.91 (0.79 to 1.05) | 0.16 | 21.2 |
| Iowa | 69 | 0.55 (0.43 to 0.71) |  | 68 | 0.58 (0.44 to 0.74) | 0.02 | 3.84 |
| Kansas | 70 | 0.72 (0.56 to 0.92) |  | 70 | 0.66 (0.52 to 0.85) | -0.06 | -7.98 |
| Kentucky | 109 | 0.66 (0.53 to 0.79) |  | 122 | 0.73 (0.60 to 0.88) | 0.07 | 9.97 |
| Louisiana | 133 | 0.88 (0.73 to 1.03) |  | 163 | 1.02 (0.86 to 1.19) | 0.14 | 15.68 |
| Maine | 20 | 0.33 (0.20 to 0.53) |  | 26 | 0.45 (0.29 to 0.69) | 0.12 | 35.4 |
| Maryland | 193 | 0.92 (0.79 to 1.05) |  | 301 | 1.34 (1.19 to 1.50) | 0.42 | 45.42 |
| Massachusetts | 99 | 0.40 (0.32 to 0.49) |  | 113 | 0.40 (0.32 to 0.49) | 0 | -0.12 |
| Michigan | 272 | 0.77 (0.67 to 0.86) |  | 275 | 0.74 (0.65 to 0.83) | -0.03 | -4.29 |
| Minnesota | 141 | 0.70 (0.58 to 0.81) |  | 186 | 0.89 (0.77 to 1.04) | 0.2 | 28.44 |
| Mississippi | 92 | 0.92 (0.74 to 1.13) |  | 102 | 1.00 (0.81 to 1.22) | 0.08 | 8.72 |
| Missouri | 128 | 0.57 (0.46 to 0.67) |  | 177 | 0.76 (0.65 to 0.89) | 0.2 | 34.78 |
| Montana | 29 | 0.67 (0.44 to 0.98) |  | 27 | 0.60 (0.39 to 0.90) | -0.07 | -11.01 |
| Nebraska | 49 | 0.73 (0.54 to 0.97) |  | 51 | 0.67 (0.49 to 0.89) | -0.07 | -8.94 |
| Nevada | 92 | 0.88 (0.71 to 1.09) |  | 126 | 1.06 (0.87 to 1.27) | 0.17 | 19.68 |
| New Hampshire | 17 | NA |  | 19 | 0.33 (0.19 to 0.54) | NA | NA |
| New Jersey | 225 | 0.71 (0.61 to 0.80) |  | 260 | 0.77 (0.68 to 0.87) | 0.06 | 8.9 |
| New Mexico | 84 | 1.10 (0.87 to 1.38) |  | 103 | 1.27 (1.03 to 1.56) | 0.17 | 15.26 |
| New York | 456 | 0.66 (0.59 to 0.72) |  | 506 | 0.69 (0.63 to 0.76) | 0.04 | 6.04 |
| North Carolina | 264 | 0.73 (0.64 to 0.82) |  | 328 | 0.83 (0.74 to 0.93) | 0.1 | 14.06 |
| North Dakota | 15 | NA |  | 16 | 0.58 (0.33 to 0.98) | NA | NA |
| Ohio | 295 | 0.72 (0.63 to 0.81) |  | 335 | 0.74 (0.66 to 0.83) | 0.02 | 3.45 |
| Oklahoma | 231 | 1.67 (1.45 to 1.89) |  | 315 | 2.19 (1.95 to 2.46) | 0.52 | 30.94 |
| Oregon | 97 | 0.63 (0.50 to 0.77) |  | 127 | 0.76 (0.63 to 0.92) | 0.14 | 21.75 |
| Pennsylvania | 255 | 0.49 (0.43 to 0.55) |  | 325 | 0.63 (0.56 to 0.70) | 0.14 | 28.21 |
| Rhode Island | 20 | 0.48 (0.29 to 0.74) |  | 21 | 0.49 (0.30 to 0.77) | 0.01 | 2.47 |
| South Carolina | 174 | 0.97 (0.82 to 1.12) |  | 209 | 1.02 (0.88 to 1.18) | 0.05 | 5.63 |
| South Dakota | 29 | 0.85 (0.56 to 1.23) |  | 23 | 0.77 (0.48 to 1.19) | -0.08 | -9.14 |
| Tennessee | 225 | 0.93 (0.81 to 1.06) |  | 290 | 1.13 (1.00 to 1.28) | 0.2 | 21.53 |
| Texas | 868 | 0.98 (0.91 to 1.04) |  | 1,050 | 1.07 (1.00 to 1.13) | 0.09 | 8.99 |
| Utah | 60 | 0.68 (0.51 to 0.87) |  | 55 | 0.56 (0.42 to 0.73) | -0.12 | -17.38 |
| Vermont | 11 | NA |  | 16 | 0.50 (0.28 to 0.89) | NA | NA |
| Virginia | 198 | 0.67 (0.57 to 0.77) |  | 267 | 0.83 (0.73 to 0.94) | 0.16 | 24.36 |
| Washington | 146 | 0.53 (0.44 to 0.62) |  | 198 | 0.73 (0.63 to 0.84) | 0.19 | 36.26 |
| West Virginia | 53 | 0.68 (0.50 to 0.90) |  | 45 | 0.56 (0.40 to 0.78) | -0.12 | -17.37 |
| Wisconsin | 138 | 0.64 (0.53 to 0.75) |  | 176 | 0.74 (0.63 to 0.87) | 0.1 | 15.65 |
| Wyoming | 14 | NA |  | 21 | 1.03 (0.63 to 1.62) | NA | NA |

Abbreviations: AAMR, age-adjusted mortality rate; SLE, systemic lupus erythematosus. NA, not available; estimates were unavailable because the CDC WONDER rate was not reported or was considered unreliable.

**Table S3. Sensitivity analysis of national systemic lupus erythematosus mortality using an underlying-cause-only definition in the United States, 1999–2023.**

| **Mortality definition** | **1999 Deaths** | **1999 AAMR (95% CI)** | **2023 Deaths** | **2023 AAMR (95% CI)** | **AAPC (95% CI)** |
| --- | --- | --- | --- | --- | --- |
| Underlying-cause-only SLE mortality | 1,316 | 0.59 (0.56 to 0.63) | 824 | 0.29 (0.27 to 0.31) | −3.23 (−4.04 to −2.54) |

Note: Deaths were included only when systemic lupus erythematosus was recorded as the underlying cause of death, defined using ICD-10 codes M32.0, M32.1, M32.8, or M32.9. Rates were age-adjusted using the 2000 U.S. standard population and are reported per 100,000 population. The underlying-cause-only analysis was conducted as a sensitivity analysis to assess whether the overall national mortality trend was robust to a narrower definition of SLE-related mortality.

Abbreviations: AAMR, age-adjusted mortality rate; AAPC, average annual percent change; CI, confidence interval; ICD-10, International Classification of Diseases, Tenth Revision; SLE, systemic lupus erythematosus.

# **Supplementary Figure:**

**Figure S1. Trends in race-specific SLE-related AAMR in the United States, 1999–2023.**

Asterisks indicate APCs significantly different from zero at P < 0.05. Abbreviations: AAMR, age-adjusted mortality rate; SLE, systemic lupus erythematosus.

**Figure S2. Trends in HHS region-specific SLE-related AAMR in the United States, 1999–2023.**

Asterisks indicate APCs significantly different from zero at P < 0.05. Abbreviations: AAMR, age-adjusted mortality rate; APC, annual percent change; SLE, systemic lupus erythematosus.

**Figure S3. Absolute change in pooled state-level SLE-related age-adjusted mortality rates between 2016–2019 and 2020–2023 in the United States.** Positive values indicate higher pooled AAMRs in 2020–2023 than in 2016–2019, whereas negative values indicate lower pooled AAMRs. Gray indicates states with unavailable or unreliable pooled AAMR estimates. Abbreviations: AAMR, age-adjusted mortality rate; SLE, systemic lupus erythematosus.

**Figure S4. Sensitivity analysis of underlying-cause-only SLE mortality in the United States, 1999–2023.** Annual age-adjusted mortality rates were calculated among decedents for whom systemic lupus erythematosus (ICD-10 codes M32.0, M32.1, M32.8, or M32.9) was listed as the underlying cause of death. Asterisks indicate APCs significantly different from zero at P < 0.05. Abbreviations: AAMR, age-adjusted mortality rate; APC, annual percent change; SLE, systemic lupus erythematosus.
